# Metagenomic assessment of the bacterial breastfeeding microbiome in mature milk across lactation

**DOI:** 10.3389/fped.2023.1275436

**Published:** 2024-07-18

**Authors:** Kelly Ingram, Collin Gregg, Allison Tegge, Jed T. Elison, Weili Lin, Brittany R. Howell

**Affiliations:** ^1^ Virginia Tech Carilion School of Medicine, Roanoke, VA, United States; ^2^ Fralin Biomedical Research Institute at Virginia Tech Carilion, Roanoke, VA, United States; ^3^ Institute for Child Development, University of Minnesota, Minneapolis, MN, United States; ^4^ Masonic Institute for the Developing Brain, University of Minnesota, St. Paul, MN, United States; ^5^ Biomedical Research Imaging Center, University of North Carolina at Chapel Hill, Chapel Hill, NC, United States; ^6^ Department of Human Development and Family Science, Virginia Tech, Blacksburg, VA, United States

**Keywords:** human milk, microbiome, breastfeeding, metagenomic, longitudial analysis

## Abstract

**Introduction:**

Research has illustrated the presence of a diverse range of microbiota in human milk. The composition of the milk microbiome varies across different stages of lactation, emphasizing the need to consider the lactation stage when studying its composition. Additionally, the transfer of both milk and skin microbiota during breastfeeding is crucial for understanding their collective impact on infant health and development. Further exploration of the complete breastfeeding microbiome is necessary to unravel the role these organisms play in infant development. We aim to longitudinally assess the bacterial breastfeeding microbiome across stages of lactation. This includes all the bacteria that infants are exposed to during breastfeeding, such as bacteria found within human milk and any bacteria found on the breast and nipple.

**Methods:**

Forty-six human milk samples were collected from 15 women at 1, 4, 7, and 10 months postpartum. Metagenomic analysis of the bacterial microbiome for these samples was performed by CosmosID (Rockville, MD) via deep sequencing.

**Results:**

*Staphylococcus epidermidis* and *Propionibacteriaceae* species are the most abundant bacterial species from these samples. Samples collected at 10 months showed higher abundances of *Proteobacteria, Streptococcaceae, Lactobacillales, Streptococcus, and Neisseria mucosa* compared to other timepoints. Alpha diversity varied greatly between participants but did not change significantly over time.

**Discussion:**

As the bacterial breastfeeding microbiome continues to be studied, bacterial contributions could be used to predict and reduce health risks, optimize infant outcomes, and design effective management strategies, such as altering the maternal flora, to mitigate adverse health concerns.

## Introduction

The in-depth characterization of the human milk microbiome, specifically the host of microbes that exist within human milk itself, is a recent development even though the presence of microbiota in human milk was identified over a half century ago (1). It was long thought that human milk was sterile, and that any microbiota present were the result of contamination from skin bacteria on the mother's nipple and breast, or from the bacteria within the infant's oral cavity (2). However, in recent years, several studies have indicated that microbes are endogenous to human milk, and that a possible internal mechanism exists by which a curated host of microbiota can become present in human milk. Although further research is needed to confirm, this proposed entero-mammary pathway suggests that microbiota from the maternal gut may travel through the maternal intestinal mucosa with assistance from dendritic cells to the lactating mammary glands. Therefore, it may be a potential source of the microbiota, including anaerobes, found in pre-colostrum and milk (3–5). Other work provides evidence that the presence of these microbiota in human milk has a beneficial impact for the infant. More specifically, evidence suggests that microbiota from human milk play a key role in seeding the gut microbiome, improving the immune system, and lowering the future risk of developing diabetes or obesity for neonates (6–10).

These studies have shown the importance of maternal microbiota transfer from breastfeeding for developing gut health, but have often failed to include a large component of the maternal bacteria typically transferred during breastfeeding. This is due to the practice of breast sanitation prior to human milk sample collection, which eliminates many of the maternal skin microbiota on the nipple and breast that are transferred during breastfeeding or during provision of pumped human milk. As of this writing, we are aware of thirteen studies that have not implemented breast sanitation prior to human milk sample collection (4, 7, 11–21). This is especially crucial because maternal skin microbiota have been associated with potential benefits to the development and health of the early infant gut microbiome, and overall health of neonates. For example, several different genera (e.g., *Propionibacterium*, *Enterococcus*, and *Streptococcus*) of microbiota typically found on the skin of humans have been linked to aiding in the development of the neonatal intestinal tract and immune system, being utilized as a dietary supplement for essential nutrients, and for playing an important role in carbohydrate metabolism (22–24).

Stage of lactation is another variable that must be considered when studying the human milk microbiome. It has been shown in previous studies that human milk microbiome composition varies across the three canonical stages of lactation: colostrum, transitional milk, and mature milk. One study found that as lactation progressed the breastfeeding human milk microbiome shifted from more maternal skin and enteric bacteria, to infant oral and skin bacteria (25). Another study showed that transitional milk and colostrum only have 48.9% of bacterial genera and 42% of bacterial species in common (26). Furthermore, the results of a study by Cabrera et al. (27) showed that colostrum was primarily composed of *Lactococcus*, *Weissella*, *Streptococcus*, *Staphylococcus*, and *Leuconostoc* species, while samples collected between 1 and 6 months post-partum had higher relative abundance of *Streptococcus*, *Prevotella*, *Veillonella*, *Lactobacillus*, *and Leptotrichia* species. Overall, these studies illustrate the need for consideration of lactation stage when analyzing human milk microbiota composition.

While it may be important to determine which microbiota are present in human milk and which microbiota are present on maternal skin independently, it is potentially more important to evaluate them collectively when trying to uncover the multiple roles these organisms play in infant development. When attempting to determine the potential benefits of microbiota transfer during breastfeeding, these bacteria are also transferred from mother to infant and comprise the complete breastfeeding microbiome. In the current study the nipple and breast were not sanitized prior to the collection of the milk sample to provide a comprehensive view of all infant bacterial exposures during breastfeeding. This provides further insight into potential impacts of breastfeeding on infant gut microbiome development, as well as other aspects of infant development influenced by the gut (i.e., immune, neural, and endocrine development) (28–30). Here we report the bacterial composition of the breastfeeding microbiome as assessed using deep metagenomic sequencing across lactation.

## Methods and materials

### Participants

Samples were collected from 15 women, who had experienced healthy pregnancies and deliveries, across lactation (see Table 1 for participant demographics) as part of the Baby Connectome Project (31) and the Baby Connectome Project—Enriched, a joint effort between the University of North Carolina at Chapel Hill and the University of Minnesota Twin Cities. All participants were enrolled at the University of Minnesota site. No women included reported having taken any antibiotics within 3 months of providing samples. Nine of the women exclusively breastfed through six months, while the other six did supplement with infant formula they still received more breast milk compared to infant formula through six months.

**Table 1:** Summary of participant demographics.

Total (*N* = 15 individuals, 46 total samples)				
Samples contributed by each participant	Delivery method		Maternal age	30.67 years
Participant 01: 4, 7, 10 months Participant 02: 4, 7, 10 months Participant 03: 4, 10 months Participant 04: 4, 10 months Participant 05: 4, 7, 10 months Participant 06: 1, 4, 7, 10 months Participant 07: 1, 4, 7 months Participant 08: 1, 10 months Participant 09: 1, 4, 7 months Participant 10: 1, 4, 7 months Participant 11: 1, 4, 7, 10 months Participant 12: 1, 10 months Participant 13: 1, 4, 7, 10 months Participant 14: 1, 4, 7, 10 months Participant 15: 1, 4, 7, 10 months	Vaginal	13 (88%)	Race	
	Emergency C-section	1 (6%)	White	14 (94%)
	Non-emergency C-section	1 (6%)	Asian	1 (6%)
	Premature		Ethnicity	
	Yes	0 (0%)	Non-Hispanic	15 (100%)
	No	15 (100%)	Hispanic	0 (0%)
	Average Birth Length	20.44 in.	Average Birth Weight	7.80417 lbs.
	Average Feed Number (At time of sample collection)		2.81579	
	Income		Education	
	25–35K	1 (6%)	Some College	2 (13%)
	50–75K	4 (27%)	College	4 (27%)
	75–100K	4 (27%)	Some Grad	2 (13%)
	100–150K	6 (40%)	Grad	7 (47%)

### Milk collection and processing

Milk was collected at the University of Minnesota when the dyad was on site for behavioral data collection, and was timed to coincide with the 2nd feed of the day whenever possible. Each participant was provided a quiet, private space equipped with a Medela Symphony hospital grade breast pump and a sterilized set of pump consumables. Mothers were asked to completely express their right breasts. Immediately following collection, the entire sample was weighed and volume recorded. The entire sample was then vortex mixed for 2 min before being aliquoted and frozen at −80°C. All samples were frozen within 30 min of the end of expression.

### DNA extraction

All steps of metagenomic analysis (including DNA extraction, library preparation, and sequencing) were completed at CosmosID, Rockville, MD (32). A 1.5 ml aliquot of untreated milk was thawed and then transferred to a 2 ml microcentrifuge tube. Each sample was centrifuged at 13,000 g, 4°C, for 20 min. The cell pellet was saved at the bottom of the tube (∼10 μl) as well as the top fat layer by carefully removing the middle liquid supernatant. 190 μl 1× PBS was added to the cell pellet and the pellet was resuspended using repeated pipetting. Twenty μl of Proteinase K was added to the resuspended cells and vortexed gently. The sample was incubated at 55°C for 18 h. Sample solution was inputted into the PowerBead Pro tube (Qiagen) and PowerSoil Pro extraction (Qiagen) was performed in accordance with manufacturer protocols. Extracted DNA samples were quantified using Qubit 4 fluorometer and Qubit™ dsDNA HS Assay Kit (Thermofisher Scientific).

### Library preparation and sequencing

DNA libraries were prepared using the Nextera XT DNA Library Preparation Kit (Illumina) and Nextera Index Kit (Illumina) with total DNA input of 1 ng. Genomic DNA was fragmented using a proportional amount of Illumina Nextera XT fragmentation enzyme. Combinatory dual indexes were added to each sample followed by 12 cycles of PCR to construct libraries. DNA libraries were purified using AMpure magnetic beads (Beckman Coulter) and eluted in QIAGEN EB buffer. DNA libraries were quantified using Qubit 4 fluorometer and Qubit™ dsDNA HS Assay Kit. Libraries were then sequenced on an Illumina NovaSeq S4 platform 2 × 150 bp (CosmosID) (32).

### Statistical analyses

CosmosID (Rockville, MD) (32) kmer based algorithms identify microorganisms based on entire genomes represented in their curated microbial genomics database, Genbook, with approximately 170,000 genomes and gene sequences. Kmers are phylogenetically stable markers identified in samples that are used in mapping to the CosmosID database. CosmosID provided filtered sequencing data, that only included high confidence calls based on proprietary filtering criteria. The Shannon alpha diversity was calculated for each sample using the Shannon diversity index in R using the microbiome package. To calculate the change in alpha diversity over time, a linear mixed effects model was used and applied in R with the following packages: microbiome, knitr, tidyr, tidyverse, ggplot2, tibble, dplyr, and nlme. Time was used as the independent variable and alpha diversity was used as the dependent variable. Each participant was included as a random effect to account for the repeated measures. Estimated marginal means (EMMs) were calculated for the model based on season (Spring, Summer, Fall, Winter) of collection averaged over the time (1, 4, 7, or 10 months) of collection to assess seasonality effects on the alpha diversity of the microbiome using the emmeans package. All statistical analyses were performed using R version 4.0.4.

A Linear discriminant analysis Effect size analysis (33), was applied to determine which abundances of microbial features significantly distinguished each stage of lactation. Processing was done using the Huttenhower lab galaxy server (https://huttenhower.sph.harvard.edu/galaxy/). Files were formatted with each column containing a group name and each line with a different level of annotation separated by “|” as is required for LEfSe input. Data was further formatted for LEfSe analysis by executing the LEfSe | Data for LEfSe function. The LEfSe | LDA Effect Size function was then selected to calculate the LDA effect size. The following parameters were used when calculating the LDA effect size Kruskal-Wallis alpha value: 0.05, Wilcoxon alpha value: 0.05, LDA score threshold 2.0, and the strategy for multi-class analysis was all-against-all. The plot of the LEfSe results was generated by executing the LEfSe | Plot LEfSe Results (34).

## Results

### Bacterial abundance

**Figure 1:**
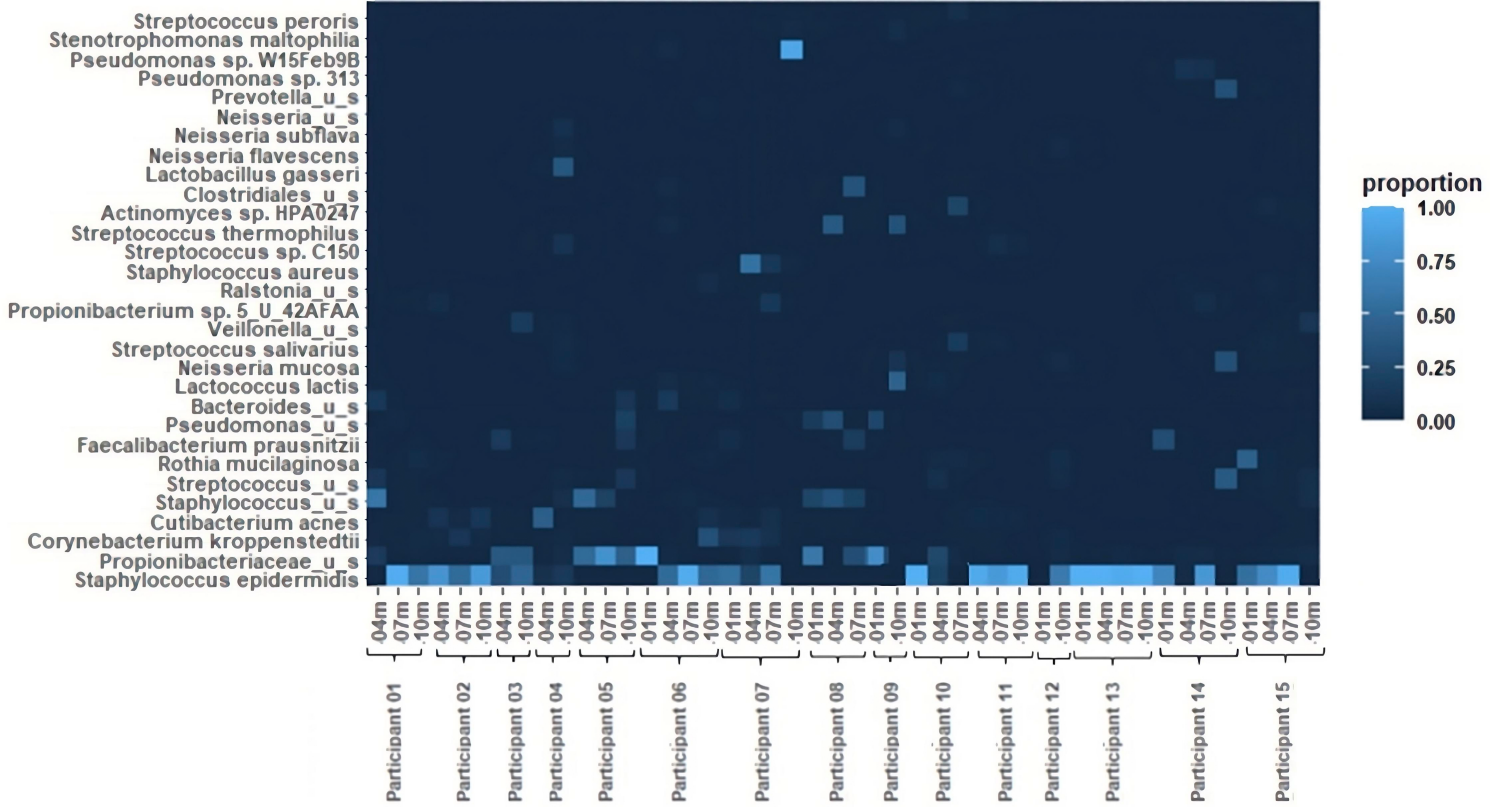
Heatmap of relative bacterial abundance. The 30 most common bacterial species in 46 breastfeeding bacterial microbiome samples from 15 participants. Samples were collected at 1 month, 4 months, 7 months, and 10 months postpartum.

The relative abundances of the 30 most common bacterial species in the 46 pumped human milk samples assessed across 15 participants are shown in Figure 1. The most abundant bacterial species across all samples was *Staphylococcus epidermidis*. This species was present in 33 of the 46 samples, and was the most abundant bacterium present in 27 of those samples. The total abundance of *S. epidermidis* ranged from 4% to 100% in *S. epidermidis* positive samples, with an average abundance of 66%. The second most abundant was *Propionibacteriaceae* unnamed species. *Propionibacteriaceae* unnamed species were found in 26 of the 46 samples and they were the most abundant in 10 of those samples. *Corynebacterium kroppenstedtii* and *Cutibacterium acnes* were each identified in 10 samples, with *C. acnes* being the most abundant bacterium in 1 sample. Additional unnamed *Staphylococcus* and *Streptococcus* species were each identified in 7 and 9 samples, respectively. *Rothia mucilaginosa* was found in 7 samples and *Faecalibacterium prausnitzii* was found in 5 samples. *Lactobacillus gasseri* was present in both samples from participant 4, with it being the most abundant bacterium at the 10-month sample collection for this participant. Other notable bacteria include *Staphylococcus aureus* and *Pseudomonas sp. W15Feb9B*. *S. aureus* was identified in 2 samples (4 m and7 m) from participant 7. The total abundance of *S. aureus* ranged from 13% (7 m) to 56% (4 m). *Pseudomonas sp. W15Feb9B* was identified in 1 sample from participant 6 with a relative abundance of 3%.

### LEfSe analysis

**Figure 2:**
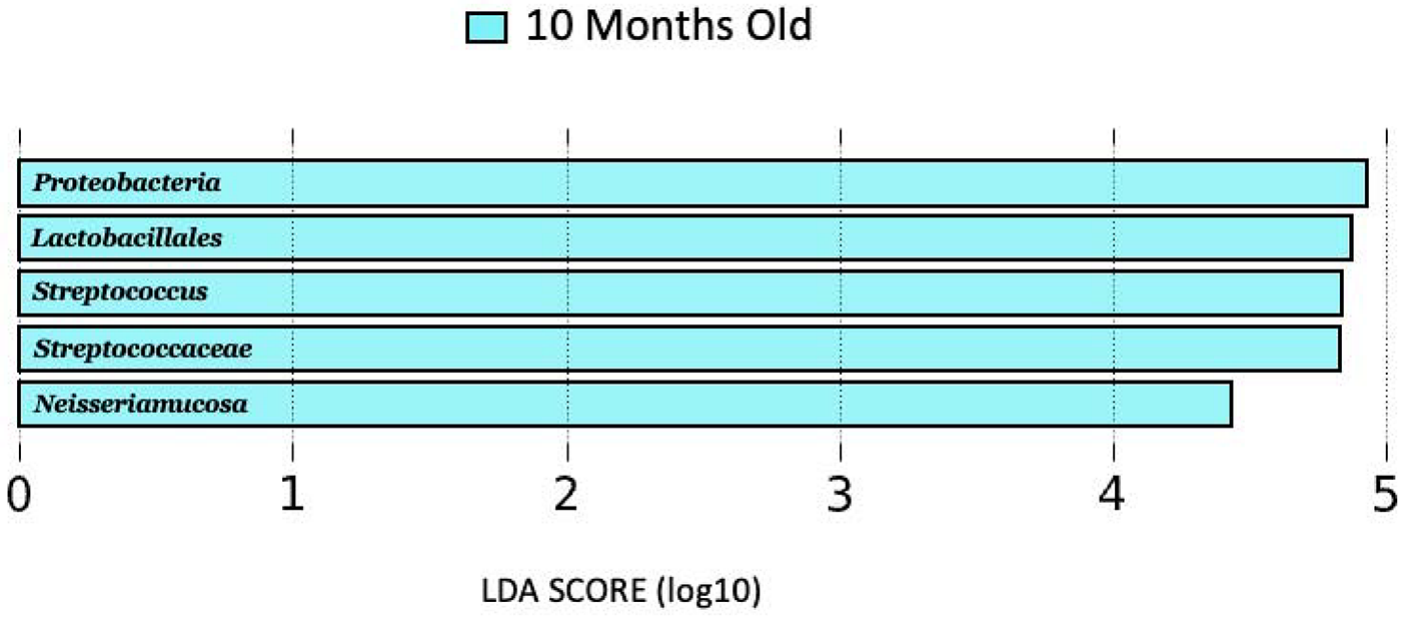
LDA score (log10) bar plot of 10 months old lactation stage, indicating a higher abundance at the 10-month-old lactation stage when compared to the 1, 4, and 7 months lactation stages.

No genera distinguished 1, 4, or 7-month milk from the other lactational stages, while 10-month milk showed higher abundances of *Proteobacteria, Streptococcaceae, Lactobacillales, Streptococcus, and Neisseriamucosa* as compared to 1-, 4-, and 7-months milk (Figure 2).

### Bacterial diversity

**Figure 3:**
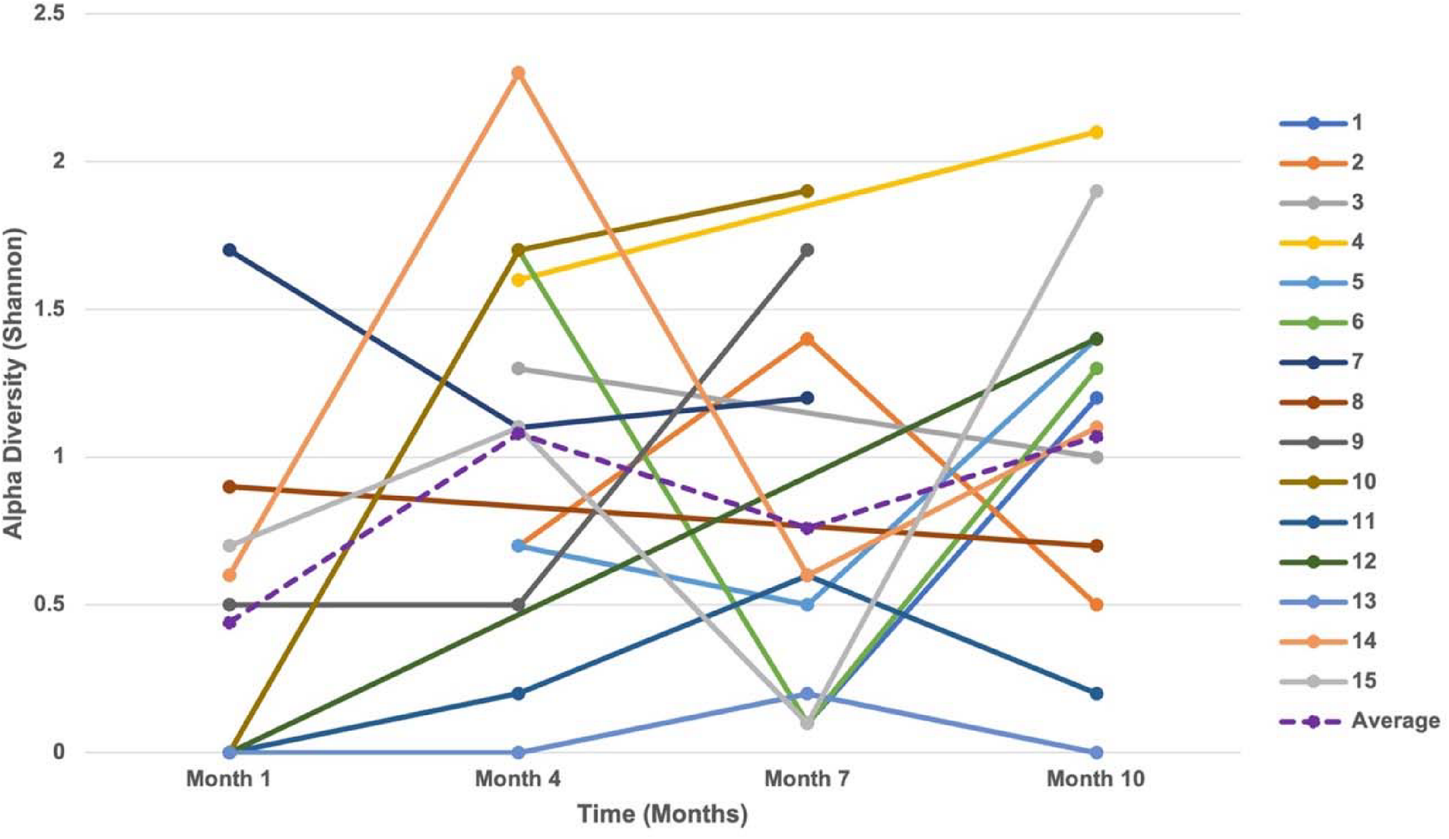
Change in alpha diversity over time. The alpha diversity (Shannon) for samples from each participant were analyzed to determine changes in bacterial diversity over time. Each color corresponds to one participant. Missing data points between two adjacent time periods are indicated by data points without a line.

The Shannon alpha diversity is shown for each sample (Figure 3). Across all the samples, the Shannon alpha diversity ranged from 0 to 2.33 and the average diversity was 0.86. The samples from participant 4 at 10 months and participant 14 at 4 months had a Shannon alpha diversity greater than 2. Each of these samples included 13 species of bacteria.

For the sample from participant 4, the most prevalent bacteria were *Lactobacillus gasseri* and *Staphylococcus epidermidis*. For the sample from participant 14, the most prevalent bacteria were *Burkholderia cepacia* and *Burkholderia cenocepacia*, which is the only occurrence of these bacteria among all samples. The alpha diversity was 0 for 7 samples (5 at 1 month, 1 at 4 months, and 1 at 10 months). The sample from participant 12 at 1 month had no bacteria present, despite no deficits in DNA extraction. For the other samples, 2 only contained *Propionibacteriaceae* unnamed species and 4 only contained *Staphylococcus epidermidis*. *S. epidermidis* was the only bacteria present in 3 of the 4 samples from participant 13.

The average alpha diversity by season was Spring (0.565), Summer (0.922), Fall (0.718), and Winter (1.229). Analysis by season showed a near significant difference (*p* = 0.0724) between the average alpha diversity in samples collected in the Spring and Winter. Differences between the average alpha diversity in the other seasons was not significant with the following *p*-values: Fall-Spring (0.9392), Fall-Summer (0.8383), Fall-Winter (0.1822), Spring-Summer (0.4458), Summer-Winter (0.6187).

### Longitudinal changes in bacterial diversity

The average Shannon diversity at 1 month was 0.44, with 5 of the samples having an alpha diversity of 0. At 4 months, the average Shannon diversity increased to 1.08. At 7 months the average Shannon diversity decreased to 0.76 and at 10 months, the average Shannon diversity increased to 1.07. From month 1 to month 4, there was an average increase in Shannon diversity of 0.64 per participant. From month 4 to month 7, there was an average decrease in Shannon diversity of 0.25 per participant. From month 7 to month 10, there was an average increase of 0.50 per participant. Overall, for the first participant sample collection (month 1 or 4) to the last patient sample collection (month 7 or 10), there was an average increase in alpha diversity of 0.52. However, for the analysis of the longitudinal changes in bacterial diversity over the study period, the results were not statistically significant at the *p* < 0.05 level (*t* = 1.862, *p* = 0.0728, Cohen's *d* = 0.25). Inverse Simpson, Gini Simpson, and Coverage bacterial diversity over time were measured using a linear effects model, but there were also no significant changes over time (Table 2).

**Table 2:** Summary of linear fixed effects analysis of changes in diversity measures over time. Shannon, Inverse Simpson, Gini Simpson, and Coverage diversity measures were used to evaluate the change in bacterial alpha diversity over time.

Diversity Measure	Estimate (β)	*p*-value
Shannon	0.05079	0.0728
Inverse Simpson	0.07998	0.2808
Gini Simpson	0.01795	0.1326
Coverage	0.03823	0.1497

## Discussion

The purpose of this study was to longitudinally assess the bacterial breastfeeding microbiome in mature milk, i.e., *all* of the bacteria that infants are exposed to during breastfeeding, not only those directly present in milk. This includes bacteria found within human milk as well as any bacteria found on the breast and nipple. Because breast sanitation was omitted from our collection procedures, the high abundance of skin bacteria was expected. *Staphylococcus epidermidis* and *Propionibacteriaceae* unnamed species are common, typically commensal, skin bacteria, and were overwhelmingly abundant in these breastfeeding microbiome samples, in agreement with past work (35). While these bacteria, along with *Corynebacterium kroppenstedtii* and *Cutibacterium acnes*, have been implicated in disease, like mastitis or acne (36, 37), there were no known instances of symptomatic disease among these participants. One participant appeared to be colonized with *Staphylococcus aureus*, which can be considerably more pathogenic and problematic for the development of childhood allergies (38). The presence of other *Staphylococcus* and *Streptococcus* species is consistent with previous human milk studies that used similar methods (3, 39). *Rothia mucilaginosa* and *Faecalibacterium prausnitzii* are also part of the normal human flora, typically colonizing the oropharynx/upper respiratory tract and the gut, respectively. Interestingly, *Pseudomonas sp. W15Feb9B* was identified in 1 sample. This bacterium was first documented in 2016 after isolation from the Ochlockonee River in Florida, USA (40). It has the potential to degrade environmental pollutants, but its role in the human microbiome is yet to be described (40).

Sample collections occurred at various times of the year based on the individual participant's post-gestational timeline. Therefore, the season (Spring, Summer, Fall, Winter) of collection was variable for each participant and the external environment could impact the diversity of bacteria present. Human milk oligosaccharides (HMOs) have been shown to fluctuate with seasonal variables, such as the weather or the dietary options available during that time of year (41). HMOs cannot be digested by infants and require bacteria to be broken down (42). If environmental factors, like changes in the season, impact the production of HMOs, it could be interconnected with fluctuations in the bacterial alpha diversity. The only relationship that came close to statistical significance was average alpha diversity in Winter vs. Spring, in which Winter was higher than the average alpha diversity in the Spring, although this relationship did not reach statistical significance at the predetermined alpha of 0.05. Since the previous study cited was conducted in Gambia, Africa (41) and our samples were collected in Minnesota, USA, the environmental factors are vastly different, but similar principles could be applied in future studies.

Sample collections also occurred across lactational stages of mature milk (1, 4, 7, and 10 months). There were no differences on a genus level between 1, 4, or 7 months. However, the 10 months lactational stage was shown to have higher abundances of *Proteobacteria, Streptococcaceae, Lactobacillales, Streptococcus, and Neisseriamucosa* as compared to the other lactational stages. This is consistent with several previous studies that showed the breastfeeding microbiome changes over lactation (27, 43, 44). There is emerging evidence that the association between breastfeeding microbiome composition and lactational stage is influenced by maternal factors such as diet, stress level, geographical location, etc. However, further studies are needed to help uncover precisely how these maternal factors influence the impact of lactational stage on the composition of the breastfeeding microbiome.

Human milk was originally thought to be sterile, but previous studies have shown that bacteria are in fact present (39); however, the diversity of bacteria is much lower than other body sites or fecal samples, for example (7). The bacterial diversity in these human milk samples is consistent with these findings. Out of the 10 samples from 1 month postpartum, 5 had an alpha diversity of 0. For each of those participants, alpha diversity either increased or stayed the same at 4 months postpartum. While the change in alpha diversity over time was not significant for this sample set, a larger sample size is needed to confirm that this null finding isn't simply due to an underpowered statistical analysis. Any future studies should incorporate a small effect size of 0.25, and a sample size of at least 24 with 4 longitudinal samples.

The maternal microbiome, whether it be vaginal, gut, skin, or milk, is crucial for shaping the infant microbiome, contributing to development and health throughout the lifespan (45). Over the years, breastfeeding rates have increased as more scientific evidence confirms the overwhelming benefits for mothers and their infants. According to the CDC's National Immunization Survey, the reported rate of ever breastfeeding increased by more than 7%, and the reported rate of breastfeeding for the first 6 months of life increased by more than 10% from 2010 to 2017 (46). As the bacterial breastfeeding microbiome continues to be studied, bacterial contributions, or the lack thereof, could be used to predict and reduce health risks, optimize infant outcomes, and design effective management strategies, such as altering the maternal flora, to mitigate adverse health concerns. In this study, some participants consistently had a low bacterial diversity, while others were richly diverse. The next questions to be answered are why do some mothers have a more diverse bacterial breastfeeding microbiome than others, and what impact will that have on their infants? It is interesting to note, that lower fecal bacterial diversity at one year of age was associated with higher cognitive performance at two years of age (47). More thorough analysis of the causes (i.e., hygiene practices, maternal health) and impacts of this bacterial composition on infant health (i.e., development of allergies or autoimmune disease) will require future, prospective, longitudinal studies.

## Data Availability

The original contributions presented in the study are publicly available. This data can be found here: https://datadryad.org/stash/dataset/doi:10.5061/dryad.rfj6q57j9.
